# Dysfunctional transcripts are formed by alternative polyadenylation in OPMD

**DOI:** 10.18632/oncotarget.20640

**Published:** 2017-09-05

**Authors:** Vered Raz, George Dickson, Peter A.C. ’t Hoen

**Affiliations:** ^1^ Department of Human Genetics, Leiden University Medical Centre, Leiden, The Netherlands; ^2^ School of Biological Science, Royal Holloway University of London, Egham, Surrey, United Kingdom

**Keywords:** PABPN1, mRNA processing, alternative polyadenylation site, autophagy, aging muscles, Gerotarget

## Abstract

Post-transcription mRNA processing in the 3’-untranslated region (UTR) of transcripts alters mRNA landscape. Alternative polyadenylation (APA) utilization in the 3’-UTR often leads to shorter 3’-UTR affecting mRNA stability, a process that is regulated by PABPN1. In skeletal muscles PABPN1 levels reduce with age and a greater decrease in found in Oculopharyngeal muscular dystrophy (OPMD). OPMD is a late onset autosomal dominant myopathy caused by expansion mutation in PABPN1. In OPMD models a shift from distal to proximal polyadenylation site utilization in the 3’-UTR, and PABPN1 was shown to play a prominent role in APA. Whether PABPN1-mediated APA transcripts are functional is not fully understood. We investigate nuclear export and translation efficiency of transcripts in OPMD models. We focused on autophagy-regulated genes (ATGs) with APA utilization in cell models with reduced functional PABPN1. We provide evidence that ATGs transcripts from distal PAS retain in the nucleus and thus have reduced translation efficiency in cells with reduced PABPN1. In contrast, transcripts from proximal PAS showed a higher cytoplasmic abundance but a reduced occupancy in the ribosome. We therefore suggest that in reduced PABPN1 levels ATG transcripts from APA may not effectively translate to proteins. In those conditions we found constitutive autophagosome fusion and reduced autophagy flux. Augmentation of PABPN1 restored autophagosome fusion, suggesting that PABPN1-mediated APA plays a role in autophagy in OPMD and in aging muscles.

## INTRODUCTION

The majority of mammalian genes have more than two PAS, and different PAS utilization generates transcripts that differ in their 3’-UTR length [1]. Alternative polyadenylation (APA) affects mRNA stability, nuclear export and hence translation efficiency [2, 3]. Genome wide studies revealed that APA utilization can be tissue and cell type-specific, and is associated with human pathologies [1, 4, 5]. APA is regulated by a dynamic protein complex, among which PABPN1 plays a central role [6, 7].

PABPN1 is a multifunctional regulator of mRNA processing including poly(A) tail length and APA utilization [8, 9]. An expansion mutation in PABPN1 (expPABPN1) is the genetic causes for Oculopharyngeal muscular dystrophy (OPMD) [10]. OPMD is a late onset myopathy where symptoms are initiated from midlife onwards and predominantly affect a subset of skeletal muscles [11]. Others and we have demonstrated that expPABPN1 leads to a widespread shift from distal to a proximal PAS utilization, resulting in increased mRNA stability and altered mRNA expression profiles that could contribute to muscle pathology [6, 12]. Reduced PABPN1 levels also increase APA utilization [6, 7, 12]. In human skeletal muscles PABPN1 levels decrease with age, and reduced PABPN1 levels have been associated with muscle weakness and muscle atrophy[13, 14]. Whether PABPN1-mediated APA utilization leads to functional transcripts that affect protein levels and muscle cell function is not fully understood.

Networks of genes of protein catabolism are most prominently dysregulated in OPMD [12, 15]. Within the catabolic protein network, clusters of the proteasome and of the autophagy related genes (ATG) stand out (Supplementary Figure 1). In previous studies we reported that the proteasome gene network is highly dysregulated in OPMD corroborated with reduced proteasomal activity [14, 16]. Autophagy is a sequential multistep process that is characterized by autophagosome formation, and subsequently autophagosome fusions with the lysosome where cytoplasmic proteins are degraded [17]. Perturbation of macroautophagy (referred here as autophagy) is a pathological signature of a wide range of pathologies including neuromuscular disorders [18, 19]. We provide the first evidence that autophagy is also affected in OPMD.

## RESULTS

### Expression of autophagy-associated transcripts is altered OPMD models

**Figure 1:**
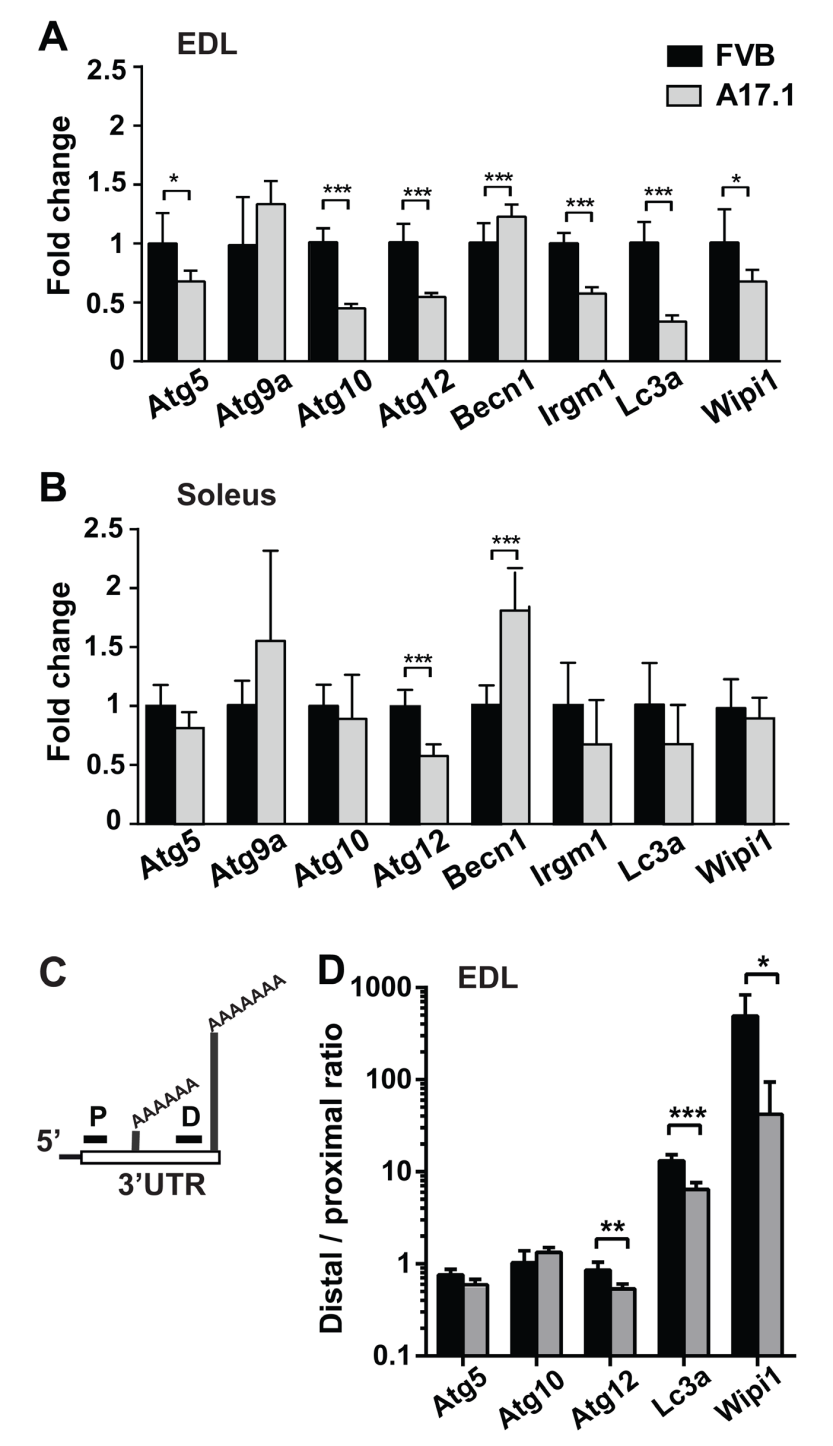
ATGs mRNA expression in the OPMD mouse model, A17.1 **A.-B.** Bar charts show results from RT-qPCR experiments of eight ATG in EDL (A) or Soleus (B) muscles of A17.1 (grey bars) and FVB control mice. Expression fold change was calculated by normalisation to *Hprt* housekeeping gene and FVB control. **C.** Schematic presentation of PCR analysis of PAS utilization in the 3’UTR. Two primer sets were used: a distal set (D) amplifies long transcripts from the distal PAS, and a proximal set (P) that additionally amplifies short transcripts with proximal PAS. APA utilization is calculated from the ratio Distal to Proximal. **D.** Bar chart shows distal to proximal ratio for five ATG in EDL. Averages and standard deviations are from five mice. Student’s T-Test was used to assess statistical significance (0.05<*p* <0.01 (*); 0.01<*p*<0.005 (**) *P* <0.005 (***).

To identify ATG candidates whose mRNA expression levels is altered in OPMD muscles, we investigated the autophagy gene network (KEGG #4140) in our previous OPMD transcriptome study [15]. KEGG #4140 was significantly dysregulated in affected muscles from OPMD patients (p-value= 0.0004), but not in muscles from pre-symptomatic human carriers of OPMD (Table 1). Of the twelve ATG whose levels were significantly altered in OPMD muscles, nine ATG were reduced in OPMD compared with age-matched controls (Table 1). Significant dysregulation of the autophagy gene network was also found in transcriptome studies from the A17.1 mouse model [12, 15]. We selected eight genes for validation whose dysregulation was consistent between microarray and RNA-seq studies [12, 15]. Expression profiles were carried out in quadriceps muscle and validation using RT-qPCR were carried out in Extensor digitorum longus (EDL) muscles. For 7 out of the 8 selected ATG genes dysregulation was validated (Figure 1A). Out of the seven genes, reduced expression levels were found in 6 genes (Figure 1A). In this mouse model, expPABPN1 is overexpressed under a muscle-specific promoter [20], but muscle atrophy is more pronounced in fast muscles compared with slow muscles [21]. Therefore, we then assessed ATG mRNA fold changes in Soleus, a slow muscle. Only two ATG displayed altered expression in Soleus (Figure 1B, respectively). Proximal PAS utilization at the 3’-UTR of transcript is prominent in A17.1 muscles [6, 12]. Five ATG (Atg5, Atg10, Atg12, Maplc3a (named here as LC3a) and Wipi1) were selected for further studies as these genes contained two distinct PASs within the 3-’UTR, and significant APA was found in A17.1 quadriceps [12]. PAS utilization was assessed by qRT-PCR using two primer sets: the distal set amplifies long transcripts from the distal PAS, and the proximal set amplifies short transcripts from proximal PAS and long transcripts. A change in the ratio between proximal to distal indicates PAS utilization indicates APA (Figure 1C, [12]). In A17.1 EDL muscle the distal to proximal ratio significantly decreased in Atg12, Lc3a and Wipi1, indicating APA (Figure 1D). In Atg5 the ratio was reduced but not significant (Figure 1D). APA in Atg10 was not found using qRT-PCR, possibly due to muscle specificity. In contrast to our previous study revealing that most APA utilization in the 3’-UTR are associated with greater transcript accumulation, for the ATG genes APA was associated with reduced expression levels. The A17.1 mouse model was generated by 30-folds expPABPN1 overexpression, and PABPN1 overexpression also affects mRNA expression levels, and molecular signatures [22, 23]. Therefore, we then assessed whether ATG mRNA processing is affected by PABPN1 levels.

**Table 1:** ATG gene dysregulation in *Vastus lateralis* muscles from pre-symptomatic and symptomatic OPMD patients.

	OPMD 60.2 ± 6.6 years Female % 66.6		Pre-symptomatic 38.5 ± 1.5 years Female % 8.3	
Gene	*p*-value	Fold change	*p*-value	Fold change
**ATG12**	0.00	-0.36	0.67	-0.06
**ULK1**	0.01	-0.51	0.27	-0.24
**ATG16L2**	0.01	-0.04	0.10	-0.04
**GABARAPL2**	0.02	-0.20	0.86	0.02
**IFNA16**	0.03	-0.03	0.83	0.01
**ATG9A**	0.03	-0.20	0.34	-0.10
**ATG4A**	0.04	-0.12	0.16	0.08
**WIPI2**	0.04	-0.02	0.84	0.01
**ATG10**	0.05	-0.18	0.97	0.00
**IFNAR2**	0.01	0.15	0.27	0.07
**ATG4D**	0.04	0.14	0.81	-0.02
**BECN1**	0.05	0.07	0.45	-0.09

P-values and fold changes (log2) were calculated from age-matched control groups using the hierarchical linear model [13], p<0.01 was set as a threshold in OPMD. For the same genes, p-value and fold change are also shown in pre-symptomatic. Per group average age ± standard deviation and % of females are indicated.

### Reduced expression of autophagy-related transcripts is mediated by PABPN1

**Figure 2:**
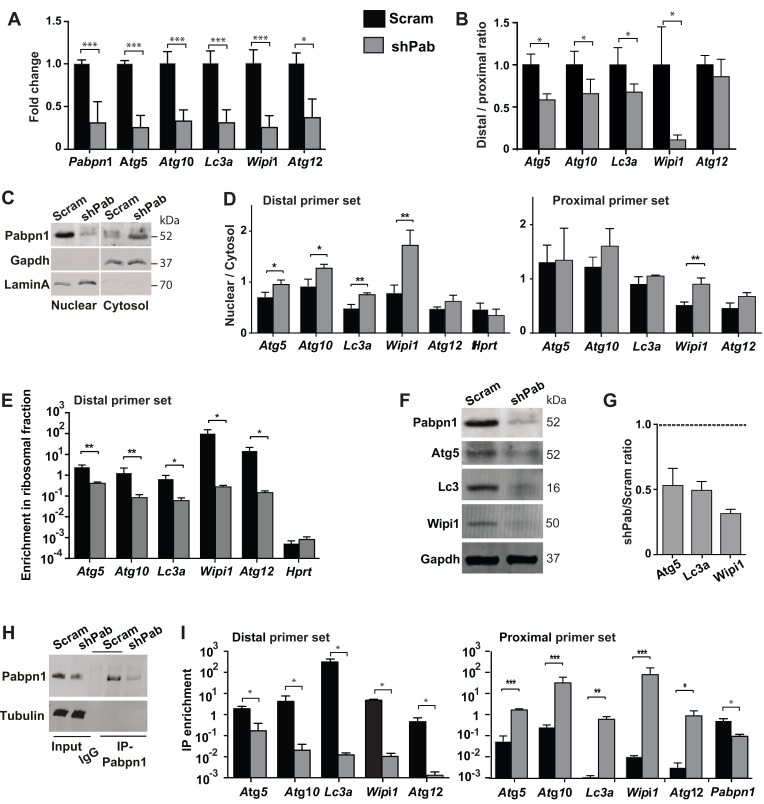
Pabpn1 regulates ATG expression through altered PAS utilization and nuclear export, affecting translation and protein abundance Experiments were performed in stable scrambled control shRNA (scram) or Pabpn1-shRNA (shPab) transfected C2C12 mouse myoblast cultures under normal growth conditions. **A.** Bar chart shows mRNA fold change of ATG. Fold change was calculated after normalization to *Hprt* housekeeping gene and control cultures. Ct values were obtained with the proximal primer set. **B.** Bar chart shows the ratio between products from distal primer and proximal primer set for five ATGs. **C.** Western blot analysis of protein lysate from cytoplasmic and nuclear fractions. Gapdh marks the cytoplasmic fraction and LaminA the nuclear fraction. **D.** Bar charts show the abundance of transcripts from distal primer set or proximal primer set between nuclear and cytosolic fractions in scram or shPab cell cultures. *Hprt* is shown as an unchanged reference gene. **E.** Bar chart shows the enrichment of transcripts from the distal primer set in the ribosomal bound fraction in scram or shPab cell cultures. *Hprt* is shown as an unchanged reference. **F.** Image shows a representative Western blot of Pabpn1, Atg3, Lc3, and Wipi1 from scram or shPab C2C12 cell cultures. Gapdh is used as loading control. **G.** Bar chart shows the ratio of protein accumulation (Atg5, Lc3a and Wipi1) between scram and shPab cell cultures. Protein abundance was normalized to Gapdh loading control. **H.** Western blot analysis of PABPN1 immunoprecipitation (IP), which was used to isolate RNA from PABPN1 complex. IP was carried out with antibodies to PABPN1 and control was carried out with beads only. Immunoblot was carried out with anti-PABPN1 antibodies and tubulin as loading control. **I.** Bar charts show the RNA-IP enrichment of transcripts from distal primer set or proximal primer set in scram or shPab cell cultures. RIP enrichment was calculated from input and *Hprt*. Averages and standard deviations are from three biological replicates. Student’s T-Test was used to assess statistical significance (0.05<*p* <0.01 (*); 0.01<*p*<0.005 (**) *P* <0.005 (***).

A stable PABPN1 knockdown (shPab) was generated in C2C12 immortalized muscle cell culture using shRNA [12]. Reduced expression of ATG genes was found in shPab culture (Figure 2A), which was associated with a lower distal to proximal ratio (Figure 2B). Since APA in the 3’-UTR of transcripts affects mRNA stability and transcript nuclear export [3], we investigated sub-cellular localization of ATG transcripts in shPab cell culture. Sub-cellular fractionation was determined by Western blot (Figure 2C) and a bioanalyzer of RNA distribution (Supplementary Figure 2). Cytoplasmic Pabpn2 accumulation in shPab was higher than in control culture (Figure 2C and Supplementary Figure 3). A change in the distribution of long transcripts was assessed by the ratio between nuclear and cytoplasmic fractions of PCR products from the distal primer set. A higher nuclear to cytoplasmic ratio was found in shPab compared with control culture (Figure 2D and Supplementary Figure 4). This indicates a higher nuclear over cytoplasmic abundance of transcripts from distal PAS. In contrast, products from the proximal primer set were generally unaffected by Pabpn1 levels (Figure 2D). Since the proximal primer set detects transcripts from distal and proximal PAS, this results suggests that transcripts from proximal PAS are more abundant in the cytoplasm. Sub-cellular localization of Hprt mRNA was not affect by Pabpn1 (Figure 2D), and therefore it was used for normalization.

Then we investigated whether Pabpn1 also affects transcript availability to the translational machinery. RNA bound to polysomes was isolated using a sucrose gradient and abundance was determined after normalization to the unbound fraction. RNA in each fraction was assessed with RNA bioanalyser (Supplementary Figure 5). ATG transcripts from the distal primer set showed reduced binding to the ribosome in shPab culture compared with the control culture (Figure 2E). As control, binding of Hprt transcript to the ribosome was unaffected by Pabpn1 levels (Figure 2E). Transcripts from the proximal primer sets (detecting both long and short transcripts) were also reduced in shPab cultures compared with controls (Supplementary Figure 6), but the effect in shPab was lower and was significant in only two genes compared with the distal primer set. For Atg5, Lc3 and Wipi1, reduced protein levels were found in shPab culture using Western blot (Figure 2F-2G). This suggest that reduced ATG expression is, in part, affected by APA utilization in the 3’-UTR.

Our results so far revealed that transcripts variants in the 3’-UTR length differ in nuclear export and hence translation efficiency and suggests a role for PABPN1. We then assessed whether transcripts variants differentially bind to Pabpn1 using RNA-immunoprecipitation (RNA-IP). RNA-IP was performed using antibodies to PABPN1 (Figure 2H), and transcripts enrichment in IP was carried out using RT-qPCR. Enrichment in IP was calculated after normalization to input and to *Hprt*. *Hprt* mRNA levels in IP were unaffected by PABPN1[12, 24]. A transcript specific enrichment in IP was calculated after normalization to Hprt and to input. We found that the abundance of ATG transcripts from distal PAS in Pabpn1-IP was lower in shPab myoblasts compared with control culture (Figure 2I). This is expected as levels of those transcripts are reduced in shPab and Pabpn1 IP in shPab is lower compared with control. However, binding of ATG transcripts from the proximal primer set to Pabpn1 was enriched in shPab culture compared with control (Figure 2I). In contrast, Pabpn1 mRNA binding to Pabpn1 protein was lower in shPab cells as expected (Figure 2I). This suggests that ATG alternative transcripts bind stronger to Pabpn1. Indeed, the ratio between proximal and distal enrichment in was close to 1000 fold higher compared with binding of transcripts from distal PAS (Supplementary Figure 7).

### Autophagy is impaired in PABPN1 down-regulated myoblasts

**Figure 3:**
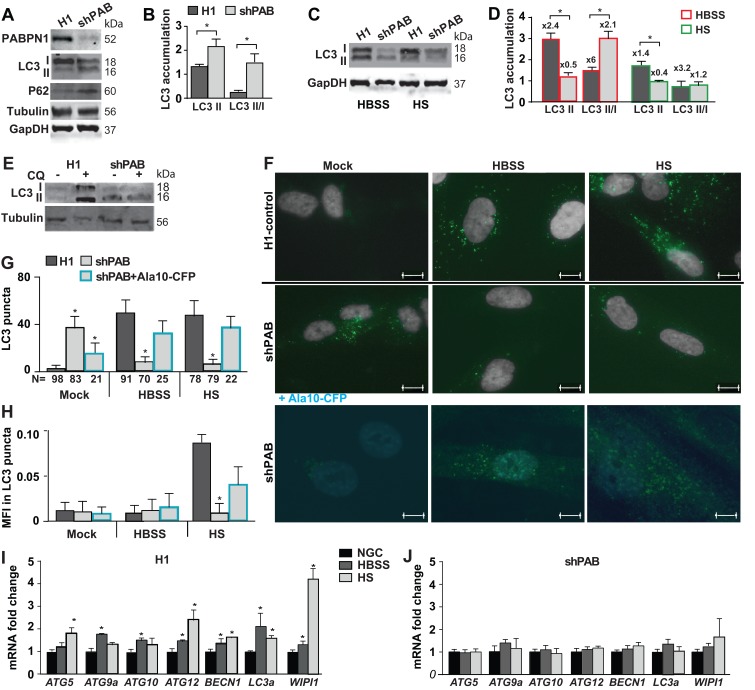
Reduced PABPN1 availability impairs autophagy in human muscle cell cultures Control (H1) or PABPN1 down regulated (shPAB) human myoblast cell cultures were incubated in normal nutrient condition (20% FBS). Stress starvation was carried out with HBSS for three hours or 0.5% HS for two days. **A.** A representative Western blot shows accumulation of PABPN1, LC3I and LC3II (18 and 16 kDa, respectively), WIPI1, and GAPDH or tubulin loading controls. **B.** Bar chart shows quantification of LC3II accumulation or LC3II/LC3I ratio. Protein levels were normalized to GAPDH. **C.** A representative Western blots shows LC3 and GAPDH loading control in starvation stressed cultures. **D.** Bar chart shows quantification of LC3II accumulation or LC3II/LC3I ratio in shPAB or control cell cultures. Protein levels were normalized to GAPDH. Fold changes from cultures in normal growth conditions are indicated above each bar. **E.** Western blot from a representative experiment shows LC3 and Tubulin protein accumulation in 20 uM chloroquine (CQ) treated cell cultures **F.** Representative images show LC3 puncta (green) in H1 control or shPab cell cultures. The nuclei are shown in grey. Scale bar is 10µm. Lower panel shows representative images of shPAB cells after transformation with PABPN1-CFP (Ala10-CFP). Ala10-CFP is depicted in cyan. Scale bar is 7.5µm. **G.** Bar chart shows quantification of mean LC3 puncta per nuclei. **H.** Bar chart shows the average of mean fluorescence intensity (MFI) of LC3 in puncta. The number of nuclei in each analysis is depicted under the X-axis. **I.**-**J.** Bar chart shows the expression of ATG transcripts in normal growth conditions (NGS) or starvation stress in H1-control (I) and shPAB (J) cultures. Fold change was calculated after normalization *HPRT* housekeeping gene and control cultures under normal nutrient condition. Standard deviation and averages are from three independent biological experiments. Student’s T-Test was used to assess statistical significance (0.05<p <0.01 (*); 0.01<*p* < 0.005 (**) *P* < 0.005 (***).

To further verify PABPN1 levels effect on ATG dysregulation we employed the human immortalized 7304.1 myoblasts, which stably expressed shRNA to PABPN1 [13]. Also in this cell model ATG expression levels were predominantly reduced by PABPN1 down-regulation (Supplementary Figure 8). We then investigated whether reduced PABPN1 levels impair autophagy. Autophagy is marked by LC3II accumulation [17]. LC3II is a proteolytic product of LC3I generated in stress conditions that induce autophagy. A larger, LC3II lipid-attached, protein product is membrane-bound marking active autophagosomes [25]. A change in the LC3II/LC3I ratio indicates autophagy. In addition, p62/SQSTM1 protein accumulation also marks autophagy [17]. In shPAB myoblast cultures p62 and LC3II levels were higher compared with the control cell culture (Figure 3A). LC3II/I ratio was also higher in shPAB cultures compared with scram cell culture (Figure 3B). This suggests that in shPAB cultures under normal growth conditions autophagosomes are constitutively formed.

We then investigated induction of autophagy in shPAB culture using starvation stress. Most often autophagy is induced by short amino acid starvation (Hanks Balances Salt Solution; HBSS), in addition muscle cell culture can be stressed for a longer period by horse serum (HS) incubation. HS leads to cell cycle arrest and cell fusion. HBSS incubation for three hours or 48 hours incubation in HS induced autophagy in control cultures marked by LC3II and an increase in LC3II/LC3I ratio (Figure 3D). In contrast, in shPAB cell culture under HBSS starvation stress LC3II level was reduced, but higher LC3II/I ratio was found due to lower LC3I level (Figure 3C and 3D). A prolonged starvation stress with HS led to reduced LC3II levels (Figure 3D). Reduced LC3 isoforms levels could result from reduced mRNA levels and reduced translation efficiency (Figure 3) and/or enhanced autophagy flux. Autophagy flux was then assessed using chloroquine (CQ) treatment, an inhibitor of autophagosomes and lysosome fusion. In control cells CQ treatment led to an accumulation of LC3II, but in shPAB it did not affect LC3 accumulation (Figure 3E and Supplementary Figure 9). This suggests impaired LC3 protein degradation via macroautophagy in shPAB cells. To confirm this, LC3 puncta (fluorescence foci), marking autophagosome formation, was quantified (Figure 3F). Consistent with LC3II accumulation, LC3 puncta formation was found in control but not in shPAB cells after HBSS or HS treatment (Figure 3G). This indicates impaired autophagosome formation is shPAB. Moreover, LC3 mean fluorescence intensity (MFI) in puncta increased in HS stress (Figure 3H) in control, indicating higher LC3 accumulation in puncta. In shPAB cells LC3 MFI was only minor increased. An increase in MFI indicates an increase in LC3 puncta size. In shPAB cells only a minor increase in LC3 MFI was found.

To confirm that autophagosome-lysosome fusion is affected by PABPN1, we restored PABPN1 levels in shPAB cultures using lentivirus transduction of particles expressing PABPN1 fused to CFP. Using a cell-based analysis in PABPN1-transduced cells we found restoration of LC3 puncta in normal growth conditions and under starvation stress (Figure 3F-3H). We chose for cell-based analysis because double transduction in the 7304.1 cell culture is not efficient. This demonstrates that in stress starvation autophagy is restored by Ala10-CFP expression.

Since PABPN1 regulates ATG expression levels, we then investigated whether autophagy activation in PABPN1 down regulated muscle cells is complemented by changes in ATG mRNAs. We found that in control cell culture ATG mRNAs were elevated by stress starvation conditions (Figure 3I). However, the induction of ATG expression was abrogated in shPAB cultures (Figure 3J). Together those data suggests that autophagy is affected by PABPN1.

### Autophagy is reduced in myotubes overexpressing expanded PABPN1

**Figure 4:**
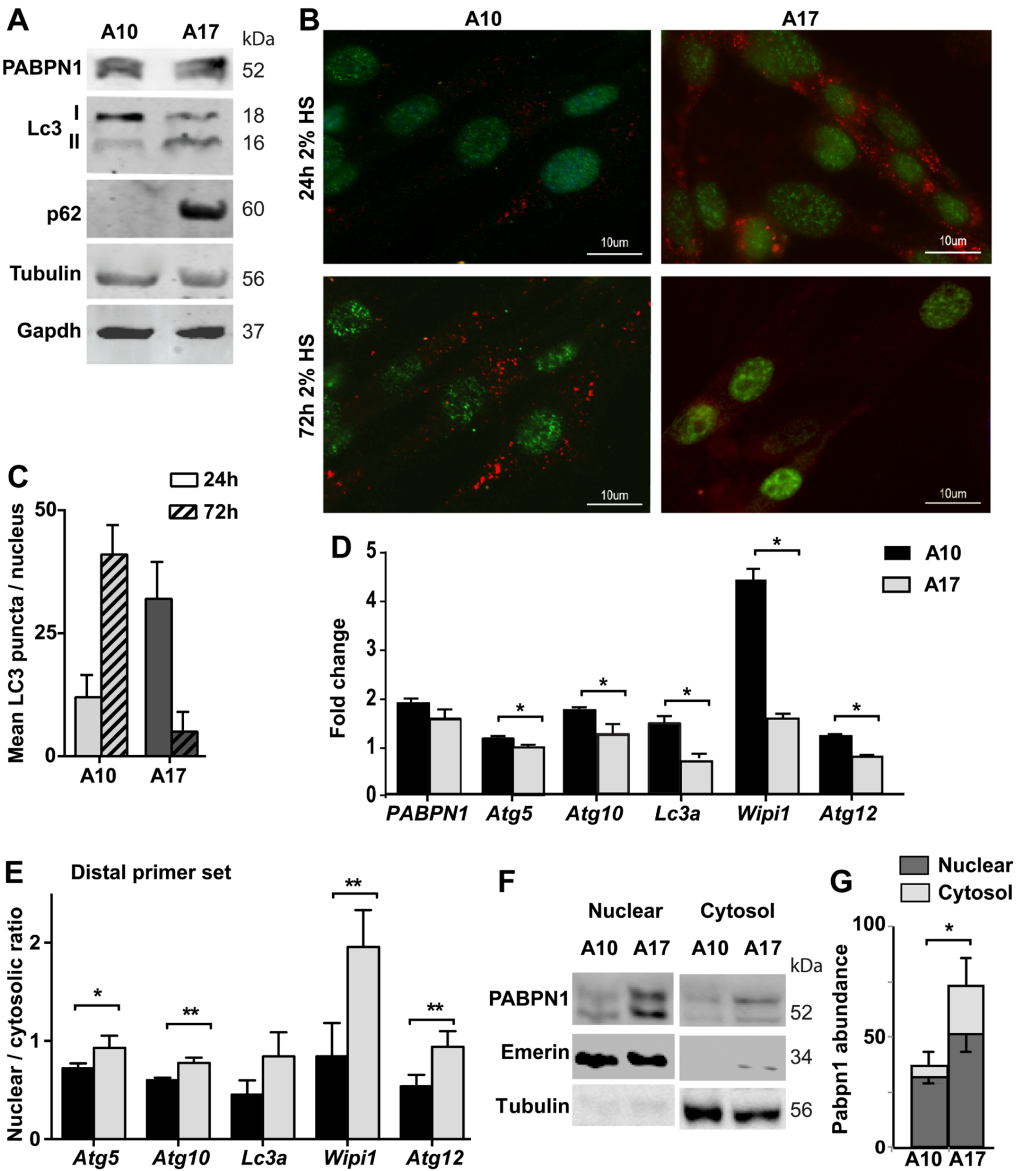
Autophagy is hyper activated in OPMD muscle cell model Experiments were performed in stable muscle cell culture over-expressing wild type PABPN1 (A10) or expPABPN1 (A17). The expression of PABPN1 transgenes was induced by incubation with 2% HS. **A.** Western blot shows levels of transgenes A10 and A17 PABPN1-FLAG (55 kDa) and endogenous Pabpn1 (52 kDa). Autophagy activation is represented by LC3II and P62. Tubulin and Gapdh are used as loading controls. **B.** Images of representative immunofluorescence with anti-FLAG (green) and anti-LC3 (red) antibodies in cell cultures that were incubated with 2% HS for 3 hours or 48 hours. Scale bar is 10 µm. **C.** Chart bar shows mean LC3 puncta per nucleus in A10 or A17 culture. Mean and standard deviations are from 100 nuclei collected from three independent experiments. **D.** Bar chart shows mRNA levels of five ATG in A10 or A17 cultures. Fold change was obtained after normalisation to *Hprt* housekeeping gene and to parental culture. **E.** Bar chart shows the nuclear to cytosolic ratio of ATG transcripts from the distal primer set. **F.** Image shows a representative Western blot of nuclear and cytosolic fractions, marked by Emerin or Tubulin, respectively. **G.** Bar chart shows PABPN1 abundance in nuclear or cytosolic fractions in A10 or A17 cell cultures. PABPN1 abundance was calculated after normalization to loading control in each fraction. Averages and standard deviations are from 4 replicates. Averages and standard deviations are from three biological independent cultures. Statistical significance is assessed by the Student’s T-Test (*p* < 0.05 is denoted with *; *p* < 0.005 is denoted with **).

We then investigated whether autophagy is also affected by the expression of expPABPN1. We employed the immortalized muscle cell (IM2), which stably expressed Ala10-PABPN1 (wild type allele; A10) or Ala17-PABPN1 (the expanded allele; A17) fused to FLAG under MyoD promoter [22]. Clones that express Ala10 or Ala17 at similar levels were selected, with 1.5-1.8 folds over endogenous Pabpn1 levels [22]. The expression of PABPN1 transgene is under Desmin promoter and induced by cell fusion [22]. Since APA is affected by cell fusion[26] and autophagy is induced in fused cultures [27], we determined expPABPN1 effect on autophagy by comparing A17 and A10 cultures. We found an increase in LC3II and p62 protein accumulation in A17 cell cultures (Figure 4A). In addition, LC3 puncta were found in A17 but not in A10 culture (Figure 4B). LC3 puncta were found in A10 culture after a prolong HS incubation (72hr), but were uncommon in A17 cultures (Figure 4B and 4C). Together, this suggests constitutive autophagy in A17 culture, but it might be blocked in fused A17.

We then investigated whether in this cell model autophagy is associated with ATG mRNA levels. RNA levels were determined in one-day fused cultures. Reduced expression levels of ATG transcripts from distal PAS were found in A17 compared with A10 cell cultures (Figure 4D). Same as in shPab culture, in A17 transcripts from distal PAS were more abundant in the nuclear fraction, compared with A10 culture (Figure 4E). Sub-cellular localization of ATG transcripts from the proximal primer set was less affected (Supplementary Figure 10). This suggests that expPABPN1 or Pabpn1 knockdown affect nuclear of ATG transcripts from distal PAS.

A previous study showed a cytoplasmic accumulation of expPABPN1 in a non-muscle cell [28]. Also in the A17 cell model we found higher expPABPN1 accumulation in A17 cytoplasmic fraction compared with A10 culture (Figure 4F-4G). Notably, in both A17 and shPab cultures cytoplasmic accumulation of PABPN1 protein concurrent with reduced abundance of transcripts from distal PAS.

## DISCUSSION

Reduced PABPN1 levels in skeletal muscles are age-associated, and correlate with muscle weakness in OPMD [13]. PABPN1 is a multifunctional regulator of mRNA processing [7]. So far, PABPN1-mediated APA in the 3’-UTR and in introns were demonstrated to be associated with OPMD [6, 12, 29]. The cellular effects of APA transcripts are not fully understood. In this study we focused on the function of transcripts generated by APA, and assessed a cellular effect, using autophagy as an example. Autophagy is reported in numerous studies to be dysregulated in neuromuscular diseases and affects muscle wasting in the elderly [18]. Here we show that APA utilization in the 3’-UTR of a subset of autophagy-related genes in OPMD is concomitant with reduced expression levels. Among those genes key regulators of autophagy, like MapLC3, were found. Reduced expression of autophagy-related genes was reported in aging tissues, and was suggested to impair autophagy [30, 31]. Our results here suggest that reduced ATGs levels are associated with autophagy. We show that ATGs levels are decreased in muscle cells with reduced PABPN1 levels, LC3II is found in normal growth condition and autophagosome-lysosome fusion is impaired under starvation stress conditions. Autophagosome-lysosome fusion can be repaired by compensation of PABPN1 levels. This observation is in agreement with our previous study in muscle cell culture showing that restoring PABPN1 levels, using PABPN1 overexpression, reverts PABPN1-mediated cell senescence [32]. A recent study further demonstrated that replacement of expanded PABPN1 with the wild type gene reverse expPABPN1-derived muscle pathology and function [33]. Our study, however, does not provide a molecular mechanism for altered autophagy in reduced PABPN1 level conditions, since PABPN1 affects APA in genes that are involved in a wide range of molecular and cellular pathways including the UPS, which also affects autophagy [34].

PABPN1-mediated APA utilization in the distal 3’-UTR has been previous demonstrated by others and us [6, 7, 12]. However, whether those alternative transcripts are functional has not been demonstrated. Functional transcripts should be exported from the nucleus and further bind to the ribosome for translation. We show that the PABPN1-dependent expression of transcripts from distal PAS is associated with reduced nuclear export. In a recent study we demonstrated that PABPN1 also plays a role in APA from intronic gene regions [29]. Also in this study we found that the default transcripts from distal PAS retain in the nucleus in muscle cells with reduced PABPN1 [29]. Here we further show that transcripts from distal PAS bind less to PABPN1 and also to the ribosome. PABPN1 shuttles between the nucleus and the cytoplasm, and thus reduced PABPN1 levels could affect nuclear export of transcript. Also in muscle cells PABPN1 knockdown caused nuclear accumulation of poly(A) RNA [35], but in HeLa and HEK293 cells PABPN1 depletion did not affect nuclear export of mRNAs [36]. This suggests that in non-muscle cells PABPN1 is dispensable for nuclear export of mRNAs, but critical in muscle cells. This is consistent with the observation that despite the ubiquitous expression of PABPN1 in every cell, symptoms are limited to muscles. Our results show that reduced nuclear export of transcripts from distal PAS is accompanied by reduced binding to PABPN1. In our experimental procedure we cannot directly measure the transcripts from proximal PAS, but a change in deltaCT between distal and proximal primer sets indicates a switch to proximal PAS, and thus confirming RNAseq studies [12]. The increase in binding of transcripts from the proximal primer set to PABPN1 in shPab cell culture suggests higher binding of short transcripts to PABPN1-complex compared with transcripts from distal PAS. Products from proximal primer set were more abundant in the cytoplasmic fraction but not in the ribosomal bound fraction, as compared with transcripts from distal PAS. This suggests that transcripts from proximal PAS are exported to the cytoplasm, maybe via binding to PABPN1-complex, but they do not bind to the ribosome, and therefore could be considered as dysfunctional transcripts. This suggests that transcripts from APA may have a lesser impact on the proteome. The fate of APA transcripts should be determined in future studies.

In the study here we focused on the effect of PABPN1-mediated APA utilization in the distal 3’-UTR. PABPN1 also affects poly(A) tail length [35], RNA decay [37] and alternative splicing, in part due to APA in introns [29]. Here we investigated the mechanism by which altered PAS in the 3’-UTR affects protein levels. Decreased expression could also be affected by altered mRNA steady-state (decay) independent of APA. The effect of additional molecular processes on the landscape of functional transcripts should be investigated in future studies.

## MATERIALS AND METHODS

### Mouse samples and cell culture

*Procedures for muscle collection and RNA extraction* from FVB and A17.1 mice are described in [21]. The A17.1 mouse model overexpressing expPABPN1 in muscle is described in [20].

Stable cell cultures of immortalised mouse myoblasts (clone IM2) over-expressing wild-type (WT) PABPN1 or expPABPN1 are described in [22]. The PABPN1 transgene expression, derived by the Desmin promoter, was induced by 4% Horse serum in DMEM. Equal cell aliquots from WT PABPN1 or expPABPN1 clones were seeded at 80% confluence, protein and RNA analysis were carried out in cultures grown with 4% HS for two days. Cell fusion does not differ between A10 and A17 cultures [22].

Stable Pabpn1 down-regulation in immortalized mouse myoblasts (C2C12) or human myoblasts (7304.1) was achieved by lentiviral delivery of shRNA constructs as described in [12, 15] respectively. All experiments in C2C12 or 7304.1 were carried out in mononucleated myoblast cultures.

### Induction of autophagy

Autophagy in C2C12 or 7304.1 cultures was induced by amino-acid starvation by incubating cultures in Hanks Balanced Salt Solution (HBSS) for 3 hours [17], in F10 supplemented with 0.5% horse serum (Invitrogen) incubation for 48 hours. Cell fusion, as marked by MF20 expression, was not found in this condition. Chloroquinone (20µM) (Sigma-Aldrich) treatment was carried out for 4 hours in growth medium. In the IM2 cell model, autophagy was induced by incubation in DMEM supplemented with 2% HS for two days.

### RT-qPCR quantification of mRNA expression and alternative polyadenylation

Total RNA was isolated from mouse muscles as previously described in Trollet et al., 2010. RNA was isolated from pelleted cell cultures using phenol extraction (Qiazol, Qiagen, Hilden, DE). cDNA synthesis was carried out using the RevertAid First Strand cDNA Synthesis Kit (ThermoScientific, MA. USA). 3ng cDNA was used as template for RT-qPCR reaction using gene specific primers (Supplementary Table 1). PCR product was detected using iQ-SYBR Green (Bio-Rad Laboratories, CA. USA). RT-qPCR was carried out using primer sets that detect long (distal PAS) or short and long (proximal PAS) transcripts. Distal PAS primers targeted sequences 5’ to the distal PAS site. Proximal primers were targeted to sequences 5’ of the proximal PAS site. APA was assessed by the ratio of power2CT values from each primer set. Although CT values are affected by primer efficiency, this can affect the ratio, but this effect is minimized when comparing the ratio between two genetic conditions. [12]. Primer sets are detailed in Supplementary Table 1. Fold change was calculated using proximal primer sets and normalised to *Hprt* gene expression. *Hprt* was selected for normalisation given its stability in stress conditions and in Pabpn1 down regulated cell cultures.

Isolation of ribosome-bound RNA was carried out in C2C12 myoblasts cultures according to the method described in [38]. Briefly, C2C12 myoblasts were grown to 80% confluence in 9 cm plates and treated for 1 hour with proliferation medium or HBSS, containing 100 µg/ml cycloheximide (CH). Cells were then washed in PBS containing 100 µg/ml CH and collected by scraping in 1ml of ice cold lysis buffer (1× solution contained 100 mM Tris, 120 mM MgCl_2_, 1.4M NaCl pH 7.4], 0.5% IGEPAL) supplemented with RnaseOUT (500 U/ml, Invitrogen), dithiothreitol (DDT) (1.5 mM), Protease Inhibitor Cocktail and CH (100 µg/ml). Protein extracts were syringed (0.33 mm, 29G needle) three times and nuclei were removed by centrifugation at 13,000 rpm for 10 min at 4°C. The supernatant was subsequently split with one half remaining undigested (input), the other half digested with RnaseI (1500U/ml, Ambion) for 30 min at room temperature, eliminating the unbound RNA. After cessation of the digestion, the lysate of both input and digested fractions was layered on frozen sucrose gradients (7–46% sucrose) and separated by ultracentrifugation at 35,000 rpm in a SW41Ti rotor (210,000 *g*, Beckmann-Coulter) for 3h at 4°C. RNA was assessed in each fraction using *RNA* kits (Agilent Genomics), and fractions containing polysomes and the unbound fractions (Supplementary Figure 4) were collected for further analysis. Proteins were then removed by proteinase K (0.15 mg/750 µl) digestion for 30 min at 42°C in the presence of 1% sodium dodecyl sulphate. RNA was extracted by acid phenol extraction and precipitation in isopropanol. Analysis of mRNA levels was carried out using RT-qPCR. The polysomal bound RNA was normalized to levels in the input (cytoplasmic fraction). RNA immunoprecipitation (RIP) and analysis of mRNA abundance in RIP were carried out as described in [12, 39]. IP enrichment was calculated by normalizing CT values in RIP to input using the distal or proximal primer set, as detailed in [12, 39].

### Protein analyses

RNA Immunoprecipitation (RIP) was performed in C2C12 myoblast cultures as described [12]. In brief, total proteins were subjected to PABPN1 immunoprecipitation using VHH-3F5, a PABPN1-specific intrabody, in the presence of RNAse inhibitor. RNA was isolated from the immune complex via phenol extraction, (Qiazol, Qiagen, Hilden, DE, USA) as above and RT-qPCR was performed as described above using primer pairs as indicated in Supplementary Table 1. RIP enrichment was calculated from input, as described in [24].

Total protein extraction was carried out using radioimmunoprecipitation assay (RIPA) buffer (20 mmol/L Tris (pH 7.5), 150 mmol/L NaCl, 5 mmol/L EDTA, 1% Nonidet P-40 (Sigma-Aldrich), and 0.05% SDS). The protease inhibitor cocktail (SigmaFAST protease inhibitor; Sigma-Aldrich) was freshly added. Samples were sonicated for 3x10 second pulses at 1MHz. Protein concentrations were measured using the Bradford assay (Bio-Rad) and 30 μg aliquots were loaded onto PAGE gels.

Subcellular fractionation was carried out as described in [40], but RNasin® Ribonuclease Inhibitor (Promega) was added for all extraction and washing buffers. Following subcellular fractionation, RNA was isolated from each fraction as described above. The nuclear fraction was verified with Lamin A/C or Emerin (1:2000; anti-Rabbit, AbCam) and the cytoplasmic fraction with Gapdh (1:10,000; anti-mouse, Sigma, MS, USA) using Western Blot. Total RNA in fractions was assessed using *Bioanalyzer RNA* kits (Agilent Genomics).

Western blot on PVDF membrane was performed using the following primary antibodies: rabbit anti-PABPN1 (1:2000 dilution; LS-B8482, LS Bio, WA, USA), rabbit anti-LC3 (1:2000 dilution; 12741, Cell Signalling, MA, USA), rabbit anti-WIPI-1α (1:1000 dilution) [41], mouse anti-SQSTM1/p62 (1:2000, ab56416, Abcam), mouse anti-tubulin (1:1000 clone DM1A, Sigma), mouse anti-GAPDH (1:10,000 dilution; G8795, Sigma, MS, USA). Blots were the incubated in secondary antibodies conjugated with IRDye 800CW or IRDye 680RD (Licor, NE, USA). Fluorescent signals were detected using the Odyssey CLx Infrared imaging system (Licor, NE, USA).

Immunofluorescence was carried out on glass-seeded cells. Cells were fixed and immunofluorescence was carried out as described in [15] using mouse-anti-LC3 (1:500) and 3F5-VHH (1:1000), visualized by rabbit-VHH (1:200) and secondary Alexa488 or Alexa594 conjugated secondary antibodies (Invitrogen). Nuclear counter-staining was achieved by adding DAPI (50 ng/ml) into the Citiflour mounting. Fluorescence was visualized with the DM5500 microscope and images were taken with the DFC360FX camera and LAS-AF version 2.3.0 software (Leica, Germany).

### Image quantification and statistical analysis

Quantification of protein accumulation from western blots was performed with ImageJ (NIH, MD, USA), values were corrected for background and normalised to GAPDH. Quantification of LC3 puncta as a measure for autophagy [17, 42] was carried out with ImageJ (NIH, MD, USA) using a standard protocol for all images: images were converted to 8-bit, LC3 puncta were segmented using a uniform threshold and particles between 10-E4 - 0.002 nm in size were filtered. The average number of puncta per cell and the mean fluorescence intensity per puncta was statistically analysed.

Statistical analyses were performed using GraphPad Prism 6 (Graphpad, CA, USA), with the unpaired Students’ T-test (assuming equal variance in all groups). Graphs and images were generated using GraphPad Prism 6 (Graphpad, CA, USA), Adobe Photoshop CS6 and Illustrator CS6 (Adobe, CA, USA).

## SUPPLEMENTARY MATERIALS FIGURES AND TABLES


