# Supplementary material for: Dysfunctional transcripts are formed by alternative polyadenylation in OPMD
Source: Oncotarget. 2017 Sep 5;8(43):73516–28. doi: 10.18632/oncotarget.20640 (PMC5650278; doi:10.18632/oncotarget.20640)
Supplement: Supplementary file 1 [file oncotarget-08-73516-s001.pdf]

Dysfunctional transcripts are formed by alternative polyadenylation in OPMD

SUPPLEMENTARY MATERIALS

Supplementary Table 1: primer sets

A. Mouse

Gene	Primer position	Forward (5'to 3')	Reverse (5'to 3')
Atg5	Proximal	AGATGGACAGCTGCACACAC	GCTGGGGGACAATGCTAATA
	Distal	ATCTCCCGTTCCTTTCCAGT	CATCGAGAGAGTGCAGCAAA
Atg9A	Proximal	TATAGGCAAGGCGGTAGCTG	GGACTATGGTAAGCCCAGCA
Atg10	Proximal	AAGCAACATCACAATCGGAG<br>T	CTATTCCAAACAGGCTTCTGC
	Distal	CGCGTGTCTGTGGTGTGTAT	AACAGAGCAGTCGTGGGTCT
Atg12	Proximal	CCCAGACCAAGAAGTTGGAA	CAGCACCGAAATGTCTCTGA
	Distal	TAGAGGAGCCTCAGCCATGT	ACGGCCCAGTTTTCAAGATA
Irgm1	Short	TGCATTCTTCCGTTTGTTGA	GGTAACCTGGCTTCTGTGGA
Maplc3a (Lc3a)	Proximal	CATGAGCGAGTTGGTCAAGA	TTGACTCAGAAGCCGAAGGT
	Distal	ATGTTCGGGTTGCTCTTTTG	TGCAGAGGAAATGACCACAG
Wipi1	Proximal	GCTCCGAGGGGAAGTTATTC	CCTCGTTCTCTCCAAAACCA
	Distal	AGATGGGTGAGGCTCCTTTT	GAGCCCAAAGGCAACTGTTA
Hprt	Proximal	CGTCGTGATTAGCGATGAT<br>G	TTTTCCAAATCCTCGGCATA
Pabpn1	Proximal	GGGCTAGAGCGACATCATGG	CAAAGCAGGTCCCTCCTCAG

B. Human

Gene	Forward (5'to 3')	Reverse (5'to 3')
ATG5	TCTGCAGTGGCTGAGTGAAC	CTTTTTCCTGTCTGGCTTGC
ATG9A	CCCTCAGGTGCACAAGGTAT	CTAGGCCCCAAATTCCTCTC
ATG10	TCATGGCTGAGCATTGTAGG	GCTGGCCAGGTAAACTCTTG
ATG12	CCCAGACCAAGAAGTTGGAA	GTCTCTTGCCACAAGCATCA
BECN1	AGCAGCTGGAGTTGGATGAC	GATTGTGCCAAACTGTCCGC
IRGM1	GCCAGCATTGGGGTATTTTA	AGTCCCCTGCCATAGTGATG
MAP1LC3a (LC3a)	CATGAGCGAGTTGGTCAAGA	TTGACTCAGAAGCCGAAGGT
WIPI1	CGCTGCGAGGAGAAGTTATT	ATTCTTCCGCCTTCCTTGTT
PABPN1	GGGCTAGAGCGACATCATGG	CAAAGCAGGTCCCTCCTCAG
HPRT	TGGTCAGGCAGTATAATCCAAAGA	TCAAATCCAACAAAGTCTGGCTTA

Supplementary Figure 1 - Gene network of protein catabolism in OPMD.

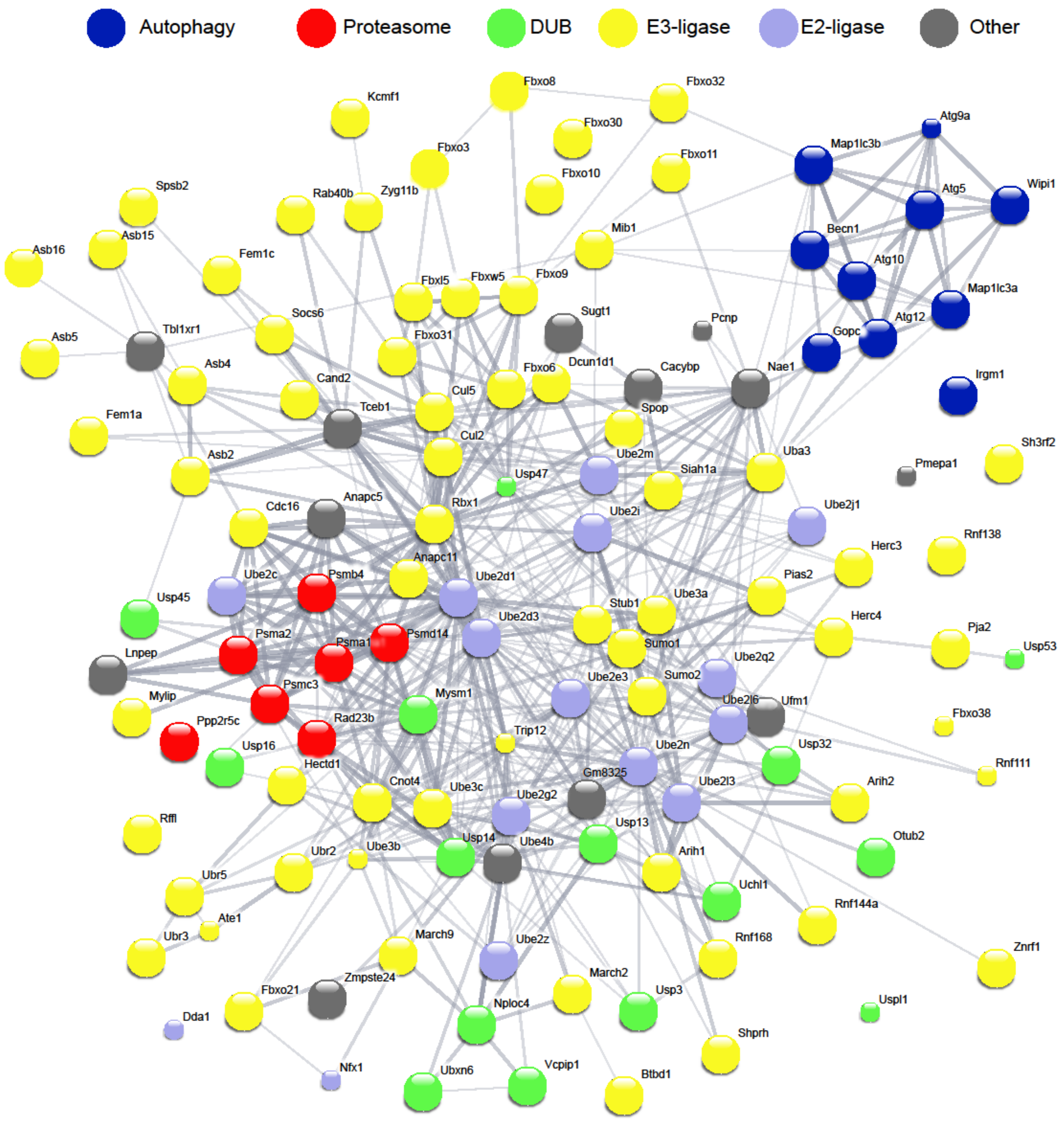

Gene network were generated from the A17.1 dysregulated genes (p<0.05; FDR; [12] using STRING online toolbox. Autophagy, proteasome, E3-ligase, E2-ligase, and deubiquitinating enzymes (DUBs) functional groups are distinguished by colors. Proteins whose function does not fall into one of the above groups are depicted in gray. Edges represent protein-protein interactions: thin lines show medium edge confidence (0.4) thick lines indicate high edge confidence (0.9).

Supplementary Figure 2: Assessment of RNA in nuclear and cytosolic fractions after subcellular fractionation using bioanalyzer.

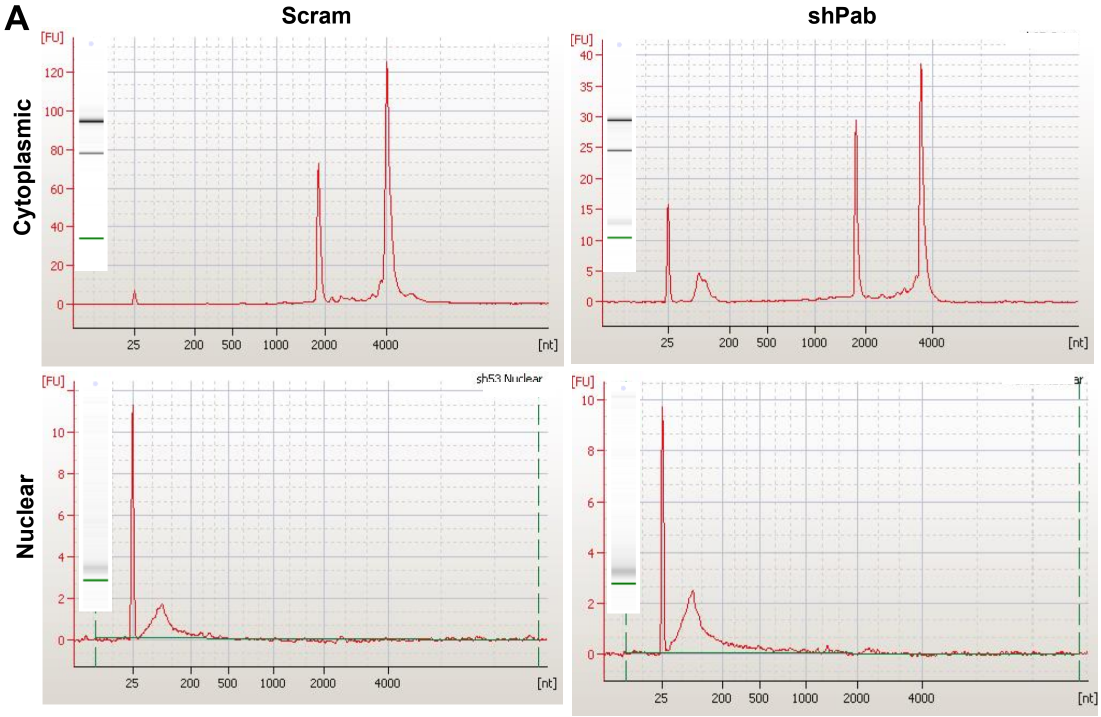

Histograms show bioanalyser outputs of RNA after subcellular fractionation. The 28S and 18S rRNA are enriched in the cytoplasmic fraction, and the nuclear fraction is rRNA depleted but enriched in tRNA. Shown are represetative images from two cultures: scram and shPab.

Supplementary Figure 3 – quantification of Pabpn1 levels in nuclear and cytoplasmic fractions.

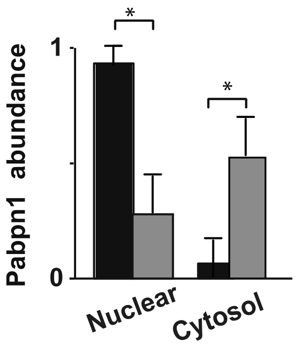

Bar chart shows Pabpn1 abundance in nuclear or cytoplasmic fraction in control (scram) and shPab C2C12 stable cell cultures. Pabpn1 levels were normalized to LaminA or Gapdh in the nuclear or cytosolic fractions, respectively. Average and standard deviation are from three independent experiments. Statistical significance was calculated with the Student's t-test; $p<0.05$ is denoted with an asterisk.

Supplementary Figure 4- qRT-PCR of mRNA levels in the nuclear fraction.

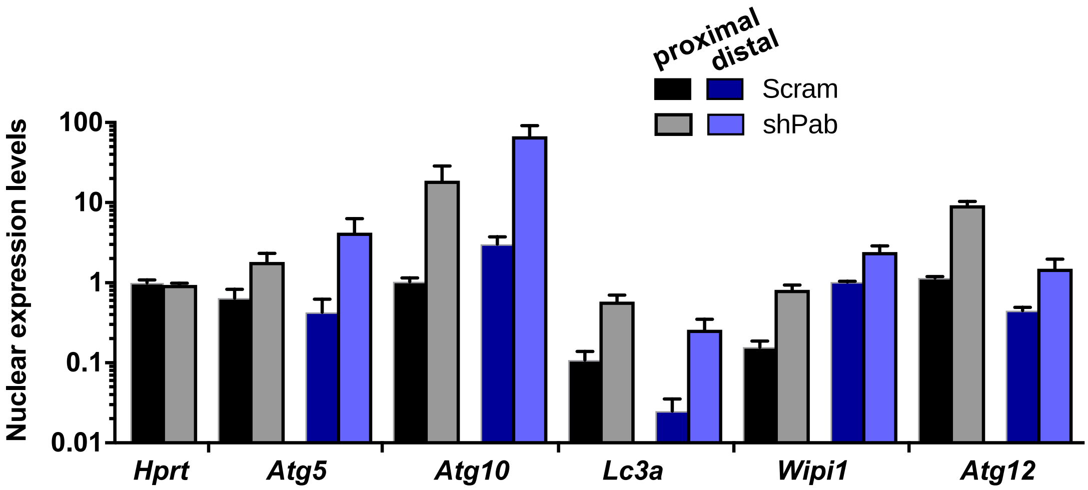

Bar chart shows *Hprt* – control and ATGs mRNA levels in the nuclear fraction. RT-qPCR was performed with distal or proximal primer set. Standard deviation and averages are from three independent biological experiments.

Supplementary Figure 5- Bioanalyzer of the ribosomal bound fraction.

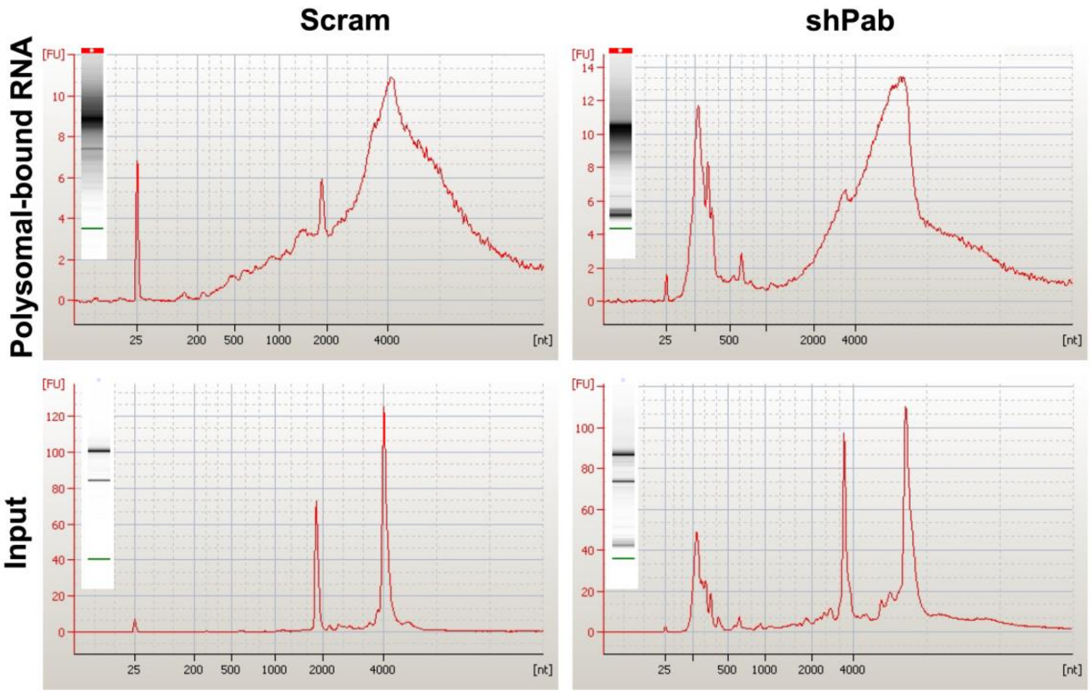

Cell lysate of the cytosolic fraction were fractionated accrding of size on a sucrose gradient. RNA in each fraction was carried out using the bionalyzer. Shown are representative plots of RNA in the polysomal fraction (ribosomal bound) and in the cytosolic input in scram and shPab culture.

Supplementary Figure 6 – qRT-PCR of mRNA enrichment in the ribosomal bound fraction using proximal primer set.

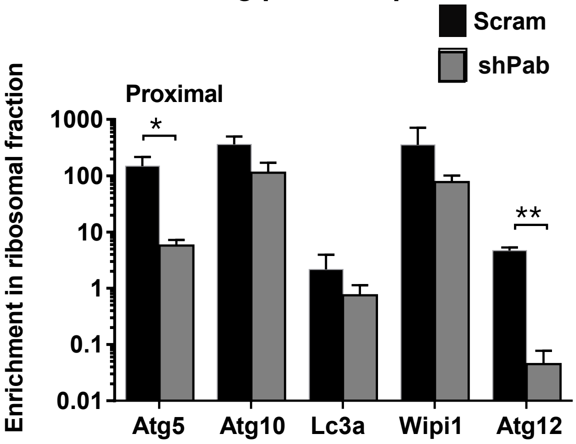

Bar chart shows ATGs mRNA enrichment in ribosomal bound fraction. RT-qPCR was performed with the proximal primer set. Enrichment was calculated from the input. Standard deviation and averages are from three independent biological experiments. Statistical significance was calculated with the Student's t-test; $p<0.05$ is denoted with an asterisk, and $p<0.001$ with two asterisks.

Supplementary Figure 7 – Ratio of ATG transcripts from proximal and distal primer sets in Pabpn1-IP.

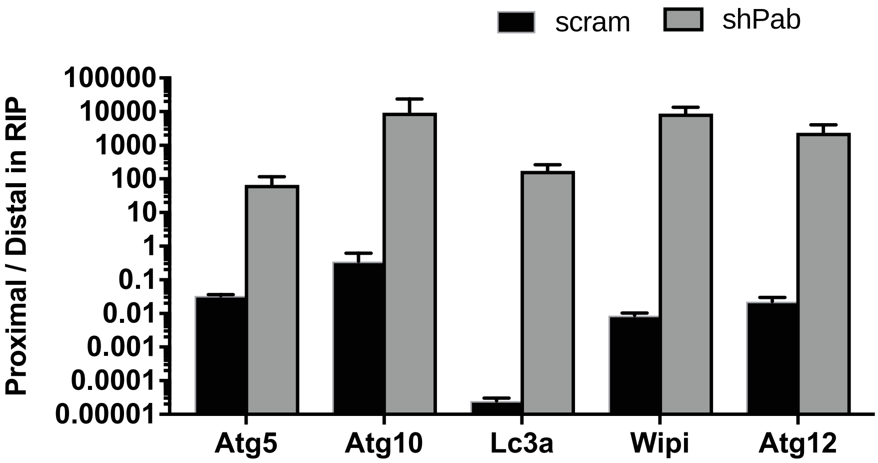

Calculation of the ratio proximal/distal in IP from experiment Figure 2I in the main text. Results show that in shPab cells ATG transcripts from proximal primer set bind stronger to Pabpn1 compared with scram.

Supplementary Figure 8 – ATGs expression is decreased in PABPN1 down regulated human myoblasts

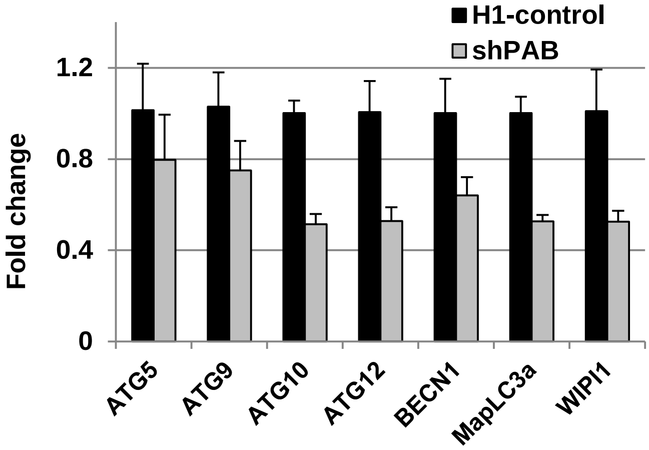

Bar chart shows ATGs mRNA expression in 7304.1 myoblasts grown under normal nutrient conditions. Fold change was normalized to HRPT in cultures grown under normal nutrient condition. Standard deviation and averages are from three independent biological experiments.

Supplementary Figure 9 – Modulation of LC3II levels by chloroquine (CQ) treatment in human muscle cell culture

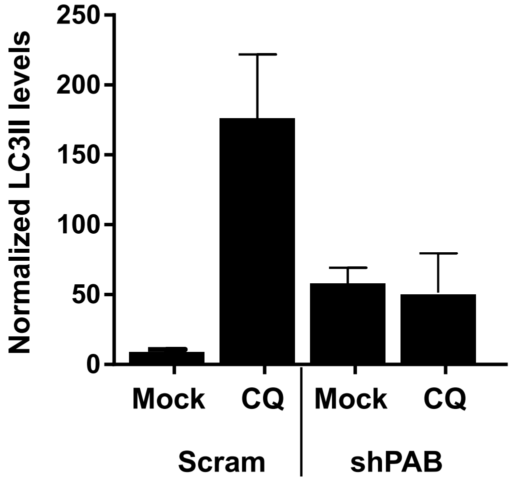

Bar chart shows levels of LC3II after normalization to tubulin. In control (Scram) LC3II levels are elevated by CQ treatment, in shPAB cell culture CQ treatment does not affect LC3II levels. Standard deviation and averages are from three biological replicates. Statistical significance was calculated with the Student's t-test; $p<0.05$ is denoted with an asterisk; NS=not significant.

Supplementary Figure 10 – Nuclear export of ATG transcripts in cell cultures expressing wild type (A10) or expanded (A17) PABPN1.

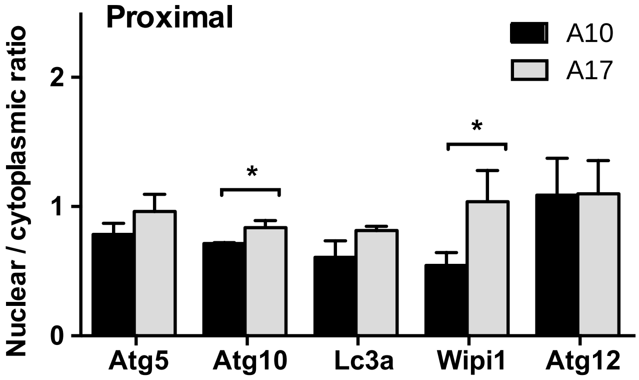

Bar chart shows the ratio nuclear to cytosolic abundance of five ATGs in A10 and A17 expressing cell cultures. RT-qPCR was performed with the proximal primer set. Standard deviation and averages are from three independent biological experiments. Statistical significance was calculated with the Student's t-test; $p<0.05$ is denoted with *.
